# Exploring the quality of life of end-stage kidney disease patients in Khartoum State, Sudan: a multicenter cross-sectional study

**DOI:** 10.1186/s12882-025-04257-2

**Published:** 2025-07-01

**Authors:** Hiba Ali Elzaki Hajomer, Osama Ahmed Elkhidir, Sara Elawad, Ahmed Balla M. Ahmed, Shaima Omer Mohamed Elawad, Mohamed H. Elbadawi, Wael Atif Fadl Elhassan, Rafa Awad Gasimelseed Mohamed, Kamil Merghani Ali, Tahani Amin Mahmoud, Sarra Mohamed Kheir

**Affiliations:** 1 Community Medicine Department, National University, Khartoum, Sudan; 2 Gulf Medical University, Ajman, UAE; 3 Faculty of Medicine, University of Khartoum, Al-Qasr Street, PO Box: 102, Khartoum, 11111 Sudan; 4 Community Medicine Department, University of Khartoum, Khartoum, Sudan; 5 National University, Khartoum, Sudan; 6 Royal College of Physicians, London, UK

**Keywords:** Health-related quality of life, End-stage kidney disease, KDQOL-SF™, Dialysis, Sudan

## Abstract

**Background:**

Given the rising incidence of end-stage kidney disease (ESKD) in Sudan, assessing health-related quality of life (HRQOL) is critical for evaluating patient outcomes. This study evaluated HRQOL and associated factors in end-stage kidney disease patients in Khartoum State renal centers in Sudan.

**Methods:**

This cross-sectional study administered the Kidney Disease Quality of Life Short Form (KDQOL-SF™) to 150 ESKD patients on maintenance dialysis for ≥ one month across 13 renal centers in Khartoum State. Data were analyzed using SPSS Statistics. Independent t-tests, ANOVA, Pearson correlation, and multiple regression analyses were conducted to assess associations. The p-value was set at 0.05 for statistical significance.

**Results:**

The Physical (40.17 ± 9.01) and Mental (47.10 ± 9.86) Component scores significantly affected HRQOL in ESKD patients. The lowest scores were observed for burden of kidney disease (31.25 ± 38) and work status (0.00 ± 50). The SF-12 Physical Component was associated with employment status (*p* < 0.001) and dialysis accompaniment (*p* = 0.011). Diabetes comorbidity affected the Mental Component (*p* = 0.017). Hospitalization frequency showed significant negative correlations with the SF-12 Mental Component (*r* = -0.249), burden of kidney disease (*r* = -0.330), effects of kidney disease (*r* = -0.303), and Kidney Disease Component Summary (*r* = -0.247). In the multiple regression model for the SF-12 Physical Component, age group was the only significant factor (*p* = 0.023).

**Conclusion:**

Both physical and mental health domains were significantly impaired in the studied ESKD population. The lowest scores were observed for disease burden and work status. Enhancing healthcare access, addressing comorbidities, and reducing financial strain may improve outcomes. Further longitudinal and case-control studies are warranted to clarify determinants of HRQOL.

**Supplementary Information:**

The online version contains supplementary material available at 10.1186/s12882-025-04257-2.

## Background

Chronic kidney disease (CKD) is defined as kidney damage or a reduced estimated glomerular filtration rate (eGFR) < 60 mL/min/1.73 m² for ≥ 3 months [1]. It is characterized by a gradual deterioration in kidney function, often necessitating treatments such as dialysis or transplantation [1, 2]. CKD is categorized into five stages on the basis of the glomerular filtration rate (GFR), with the 5th stage representing end-stage kidney disease or kidney failure, where the GFR is less than 15 ml/min [2, 3]. Patients with end-stage kidney disease have higher mortality rates, ranging from 20% to 50% over 24 months, even with timely dialysis [3]. Worldwide, it is estimated that between 4.902 and 9.701 million people suffering from end-stage kidney disease (ESKD) need renal replacement therapy (RRT), with a higher prevalence observed in low- to middle-income nations [4]. The prevalence of the disease is notably high in African nations, affecting approximately 8–16% of the population [5]. The lack of trustworthy health information systems has made it difficult to determine the exact prevalence of ESKD in the area. According to statistics from 13 sub-Saharan nations, the overall prevalence of CKD was estimated to be 13.9% [6]. In Sudan, access to kidney replacement therapy is constrained and not universally available. Governmental and private hemodialysis centers serve 62.6% and 37.4% of the dialysis population, respectively [7]. Patients often incur substantial out-of-pocket expenses, with the median annual direct per capita cost of ESKD reaching 38,600 SDG ($1,723.2) in Khartoum State [8]. 

Health-related quality of life (HRQOL) is a multifaceted concept that encompasses a patient’s overall assessment of how their health condition and medical interventions influence their physical, mental, and social dimensions of well-being [9]. ESKD patients report poorer HRQOL compared to the general population [5, 10]. Numerous studies have identified various determinants that contribute to and impact health-related quality of life (HRQOL) among individuals suffering from ESKD. Patients with ESKD commonly experience constrained functional status across domains such as physical, role, social, and mental functioning due to the manifestations of the disease and the associated treatment protocols [11, 12]. Multiple comorbidities, lower albumin and haemoglobin levels, and other clinical factors have been consistently linked to a reduced quality of life. Additionally, factors such as age, gender, living situation, and income have been linked to HRQOL [13].

Research on quality of life among ESKD patients in Sudan is limited. A recent study of 168 patients found that transplant recipients had significantly better quality of life than those on dialysis, highlighting the strong impact of treatment type on well-being [14].

Despite Sudan’s growing ESKD burden, gaps persist in understanding HRQOL determinants. This highlights the need for further exploration of patient-centered outcomes in this population. Assessing health-related quality of life (HRQOL) has become crucial and is often necessary for evaluating health outcomes [11]. Evaluating HRQOL in patients with ESKD could provide a valuable way to understand the impact of healthcare interventions in situations where a cure is not feasible. Therefore, this study aimed to assess health-related quality of life and its associated factors in adult patients with end-stage kidney disease who were attending public specialized renal centers in Khartoum State, Sudan.

## Methods

### Study design and setting

This was a descriptive, cross-sectional study conducted at the health facility level in January 2020. It was carried out in 13 selected dialysis centers in Khartoum State, Sudan. These centers provide dialysis services to registered renal patients and handle emergency cases. Out of 32 renal centers in Khartoum State, only 13 centers were included, as they were the ones where patients attended follow-up visits during the research period. The renal centers included in the study were Alwaledeen, Tropical Disease, Alnaw, Ombada, Chinese Friendship, Military Hospital, Academy, Elshahida Salma, Bashaier, Ibnsina, Elsafia, Renal Transplant Association, and Ahmed Gasim. We used strengthening the reporting of observational studies in epidemiology (STROBE) reporting guidelines for cross-sectional studies to ensure proper reporting for the study [15].

### Study participants

The study included adult male and female patients aged ≥ 18 years who were diagnosed with ESKD and had been on maintenance hemodialysis for at least one month. This minimum duration was chosen to ensure that participants had sufficient experience with dialysis to meaningfully assess its impact on their quality of life.

Exclusion criteria were patients with acute or severe health conditions that could impair communication and those who declined participation. These exclusions aimed to ensure valid and complete responses during data collection.

### Sample size and sampling technique

The required sample size was calculated using Cochran’s formula for single population proportion [16], with a 95% confidence interval, a proportion of 0.001 (based on the global prevalence of ESKD in 2019) [17], and an accepted sample error of 0.05.

Equation:

$$\:{n}_{0}=\frac{{t}^{2}pq}{{d}^{2}}$$

 n = sample size.

t = standard error associated with the chosen level of confidence (%95).

p = proportion of the population (prevalence).

q = 1-p.

d = accepted sample error (reduced to 0.005 to increase precision in estimating a rare outcome).

Based on this calculation, the final sample size was 154 patients. Ultimately, data were collected from 150 patients. The sample was distributed proportionally on the basis of the average patient frequency at each facility, with the interval for each center determined as shown in Table S1. Systematic random sampling was used within each center. Random days of the week were selected, and on each selected day, the patient list was used as the sampling frame. Patients were selected at fixed intervals as they arrived until the desired number was reached.

### Study tools

A face-to-face interview questionnaire was conducted by trained medical students. Informed consent was obtained immediately before each interview. Patients’ medical records were reviewed to extract clinical and sociodemographic data. The sociodemographic variables included age, gender, residence, education level, and employment status. Medical information covered the impact of kidney failure on daily activities, comorbidities, history and duration of renal impairment, number of dialysis locations attended, frequency of dialysis sessions per week, accompaniment during dialysis visits, presence and type of health insurance, and history of hospitalization. We used the standardized Kidney Disease Quality of Life Short Form (KDQOL-SF™), version 1.3, to assess the quality of life in these patients [18, 19]. This tool is specifically designed for individuals with kidney disease and those undergoing dialysis. It includes 43 items targeting kidney disease and 36 items that form a generic core, along with an overall health rating item. The questionnaire consists of 80 items divided into 19 dimensions. The disease-specific Component of KDQOL-SF™ 1.3 comprises 43 items across 11 domains, including the symptom/problem list (12 items), effects of kidney disease (8 items), burden of kidney disease (4 items), cognitive function (3 items), quality of social interaction (3 items), sexual function (2 items), sleep (4 items), social support (2 items), work status (2 items), patient satisfaction (1 item), and dialysis staff encouragement (2 items).

The SF-36 Component contains 36 items that assess eight domains of functioning and well-being on a 100-point scale. These domains include physical function (10 items), role limitations due to physical problems (4 items), role limitations due to emotional problems (3 items), pain (2 items), general health perceptions (5 items), social function (2 items), emotional well-being (5 items), and energy/fatigue (4 items). The final item, an overall health rating, asks respondents to rate their health on a 0–10 scale. Results from the SF-36 are further summarized into a Physical Component Summary (PCS) score and a Mental Component Summary (MCS) score. PCS aggregates items from physical function, role physical, pain, and general health, while MCS aggregates items from role emotional, emotional well-being, energy, and social function [18]. According to Mapes et al. [20], the kidney disease-targeted scale items are also summarized into a Kidney Disease Component Summary (KDCS) score on a 100-point scale.

The standard KDQOL-SF™ 1.3 scoring program, based on a Microsoft Excel 97 spreadsheet, includes detailed computation methods. Scores for each dimension range from 0 to 100, with higher scores indicating better health-related quality of life (HRQOL). The health change (question 2) of the SF-36 and the 0–10 overall health rating (question 22) are scored as single items [18]. The KDQOL-SF™ 1.3 questionnaire was administered in a single session, typically taking around 16 min, with the entire questionnaire, including this section, requiring approximately 20 min. To minimize patient burden, interviews were conducted during dialysis sessions or at times convenient for the participants.

### Data analysis

The Statistical Package for the Social Sciences (SPSS) version 26 was used for data entry and statistical analysis. Frequency tables and percentages were used to present the categorical variables. Means, standard deviations, medians, and interquartile ranges were used to describe the continuous data. Normality tests were applied to all scales. All scales exhibited non-normal distributions, except for the SF-12 Physical Health and SF-12 Mental Health composite scores, which demonstrated normal distribution. Independent t tests and analysis of variance (ANOVA) were used to determine the differences across socio-demographic and clinical data regarding the SF-12 scales, as they were normally distributed. Mann-Witney t test and Kruskall Walis test were used for Kidney Component Scale as it was non normally distributed. Pearson’s and Sperman’s correlations were used to assess the association between total cost, hospitalization time, and duration of hospitalization with quality of life scales. Multiple linear regressions were used to find associations between the study variables. The used level of significance was 0.05.

## Results

### Sociodemographic, clinical, and dialysis-related characteristics

A total of 150 patients participated in the study, with a response rate of 97%. The median age was 48 years, and the most common age group was 51–60 years, representing one-quarter of the participants. The majority—almost two-thirds—were male. Most of them (90.7%) lived in urban areas. Almost 36% were working at the time, even though nearly two-thirds of the sample reported having stopped their daily activities. The majority (70.7%) had hypertension as comorbidity with kidney failure. Nearly one-third of the patients (34.2%) had visited more than one dialysis center. Almost 60% of the participants went to dialysis accompanied. The majority of the sample (85.3%) had health insurance (Table 1).

**Table 1:** Sociodemographic, clinical, and dialysis-related characteristics of the participants (*N* = 150)

Variables	Frequency	Percentage (%)
**Age:**		
21–30	30	20.0%
31–40	22	14.7%
41–50	30	20.0%
51–60	39	26.0%
61–70	26	17.3%
> 70	3	2.0%
**Gender**:		
Male	92	61.5%
Female	58	38.5%
**Current place of residence**:		
Urban	136	90.7%
Urban slum	9	6.0%
Rural	5	3.3%
**Highest level of education**:		
Not attended/illiterate	11	7.5%
Primary	36	24.5%
Secondary	60	40.8%
Graduate	29	19.7%
Postgraduate	10	6.8%
Other types of education	1	0.7%
**Current employment status**:		
Currently working	54	36.5%
Not working currently	94	63.5%
**Have you stopped your daily life activities due to kidney failure?**		
Yes	83	67.5%
No	40	32.5%
Presence of comorbidities*:		
Hypertension	106	70.7%
Diabetes	23	15.3%
Autoimmune disease	2	1.3%
Other	18	12.0%
**Do you have any history of renal impairment**:		
Yes	72	48.0%
No	78	52.0%
**Duration of kidney failure**:		
Less than 1 year	9	6.0%
1–3 years	77	51.3%
4–6 years	32	21.3%
7–10 years	21	14.0%
More than 10 years	11	7.3%
**Number of places of dialysis**:		
One place	98	65.8%
Two places	23	15.4%
Three places	14	9.4%
More than three places	14	9.4%
**Times per week for dialysis**:		
1 time	1	0.7%
2 times	130	86.7%
3 times	19	12.7%
**Go to dialysis accompanied**		
Yes	89	59.7%
No	60	40.3%
**Presence of health insurance**		
Yes	128	85.3%
No	22	14.7%
**Type of insurance**:		
Social health insurance	118	92.2%
Private insurance	9	7.0%
Other	1	0.8%
**Have you been hospitalized before or after the beginning of dialysis**:		
Yes	91	60.7%
No	59	39.3%

*Participants were allowed to select more than one choice

### Physical and mental functioning

A univariate analysis of sociodemographic, clinical, and dialysis characteristics in relation to the Physical Component scale was conducted. Patients who were working at the time reported significantly lower physical functioning (mean = 44) compared to those in the non-working group (p-value < 0.001). Additionally, patients who went to dialysis accompanied had significantly lower physical functioning (mean = 39) compared to those who had no one (p-value = 0.011) (Table 2). Regarding the Mental Component scale, patients with diabetes had significantly better mental functioning than those without diabetes (mean = 51 vs. 46; p-value = 0.017) (Table 3).

**Table 2:** Univariate analysis of sociodemographic, clinical, and dialysis-related characteristics of the participants and the SF-12 physical component (*N* = 150)

Variables		SF-12 Physical Component		
		Mean	Standard deviation	P-value
Age groups	21–30	43	8	0.062
	31–40	44	8	
	41–50	39	9	
	51–60	39	9	
	61–70	37	10	
	> 70	35	3	
Gender	Male	41	8	0.201
	Female	39	10	
Residency	Urban	40	9	0.451
	Urban slum	38	9	
	Rural	45	8	
Educational level	Not attended/illiterate	39	7	0.270
	Primary	38	9	
	Secondary	42	10	
	Graduate	41	8	
	Postgraduate	38	10	
	Other type of education	33		
Are you currently working?	Currently working	44	8	0.000**
	Not working currently	38	9	
Have you stopped working due to kidney failure?	Yes	40	9	0.642
	No	41	11	
Presence of comorbidities^a^	Hypertension	39	9	0.097
	Diabetes	38	8	0.268
	Autoimmune disease	31	6	0.161
	Other diseases	39	9	0.731
Do you have any history of renal impairment?	Yes	39	9	0.208
	No	41	9	
Duration of kidney failure in years	Less than 1 year	44	7	0.278
	1–3	39	9	
	4–6	43	10	
	7–10	40	9	
	More than 10 years	37	7	
How many places to receive dialysis?	One place	39	9	0.565
	Two places	41	9	
	Three places	43	8	
	More than three places	40	8	
Times per week to receive dialysis	1 time	32		0.608
	2 times	40	9	
	3 times	41	12	
Go to dialysis accompanied	Yes	39	9	0.011*
	No	43	8	
Presence of health insurance	Yes	40	9	0.421
	No	39	9	
Type of insurance	Social health insurance	41	9	0.259
	Private insurance	37	6	
	Other			
Have you been hospitalized before or after the beginning of dialysis?	Yes	39	9	0.104
	No	42	9	

^a^ Participants were allowed to select more than one choice, which is why the analysis was performed as 0/1 for each choice

*p-value < 0.05

**p-value < 0.001

**Table 3:** Univariate analysis of sociodemographic, clinical, and dialysis-related characteristics of the participants and the SF-12 mental component (*N* = 150)

Variables		SF-12 Mental Component		
		Mean	Standard Deviation	p-value
Age groups	21–30	45	10	0.341
	31–40	46	8	
	41–50	49	9	
	51–60	47	12	
	61–70	47	7	
	> 70	52	4	
Gender	Male	47	10	0.600
	Female	48	10	
Residency	Urban	46	10	0.068
	Urban slum	53	8	
	Rural	54	11	
Educational level	Not attended/illiterate	50	5	0.391
	Primary	47	11	
	Secondary	46	10	
	Graduate	50	10	
	Postgraduate	43	7	
	Other type of education	55		
Are you currently working?	Currently working	48	10	0.684
	Not working currently	47	10	
Have you stopped working due to kidney failure?	Yes	46	10	0.225
	No	49	10	
Presence of comorbidities^a^	Hypertension	47	10	0.821
	Diabetes	51	7	0.017*
	Autoimmune disease	42	1	0.481
	Other diseases	49	6	0.240
Do you have any history of renal impairment?	Yes	46	10	0.178
	No	48	9	
Duration of kidney failure in years	Less than 1 year	42	12	0.682
	1–3	47	9	
	4–6	48	10	
	7–10	45	12	
	More than 10 years	48	9	
How many places to receive dialysis?	One place	47	11	0.818
	Two places	47	8	
	Three places	47	8	
	More than three places	50	8	
Times per week to receive dialysis	1 time	44		0.837
	2 times	47	10	
	3 times	48	8	
Go to dialysis accompanied	Yes	47	11	0.715
	No	47	9	
Presence of health insurance	Yes	47	10	0.755
	No	46	10	
Type of insurance	Social health insurance	47	10	0.824
	Private insurance	46	7	
	Other			
Have you been hospitalized before or after the beginning of dialysis?	Yes	47	10	0.445
	No	48	10	

^a^Participants were allowed to select more than one choice, which is why the analysis was performed as 0/1 for each choice

*p-value < 0.05

### Kidney disease component summary (KDCS)

A univariate analysis was performed regarding patient characteristics against KDCS. Compared with people living in urban areas, those living in rural areas reported a significantly higher quality of life using the KDCS (p-value = 0.037). Additionally, patients who had stopped working experienced a significantly better quality of life than those who were working at the time of the study (p-value = 0.011). Patients who did not have hypertension had a significantly better quality of life (median = 67.61 vs. 58.3) than those who did (p-value = 0.014). Having health insurance was also significantly associated with a better quality of life (p-value = 0.001), whereas going to dialysis accompanied was significantly associated with a poorer quality of life (p-value = 0.002) (Table 4).

**Table 4:** Univariate analysis of sociodemographic, clinical, and dialysis-related characteristics of the participants and kidney disease component summary (*N* = 150)

Variables		Kidney Disease Component Summary		
		Median	Interquartile range (IQR)	p-value
Age groups	21–30	56.72	20.74	0.657
	31–40	62.89	16.25	
	41–50	62.61	22.36	
	51–60	59.30	18.09	
	61–70	60.54	18.12	
	> 70	53.24	20.38	
Gender	Male	62.96	19.50	0.476
	Female	58.81	17.99	
Residency	Urban	58.74	17.59	0.037*
	Urban slum	65.18	11.86	
	Rural	72.29	2.84	
Educational level	Not attended/illiterate	60.02	18.69	0.092
	Primary	55.89	16.22	
	Secondary	63.03	16.21	
	Graduate	59.30	18.69	
	Postgraduate	66.57	34.22	
	Other type of education	71.65	0.00	
Are you currently working?	Currently working	61.69	20.80	0.505
	Not working currently	59.45	15.95	
Have you stopped working due to kidney failure?	Yes	58.30	18.22	0.011*
	No	66.32	20.15	
Presence of comorbidities^a^	Hypertension	58.30	17.48	0.014*
	Diabetes	60.02	20.88	0.425
	Autoimmune disease	53.75	5.08	0.366
	Other diseases	59.30	18.30	0.692
Do you have any history of renal impairment?	Yes	59.09	17.35	0.674
	No	60.48	19.33	
Duration of kidney failure in years	Less than 1 year	54.62	25.17	0.101
	1–3	57.89	17.94	
	4–6	66.27	15.29	
	7–10	59.60	17.02	
	More than 10 years	58.88	21.01	
How many places to receive dialysis?	One place	61.74	19.11	0.387
	Two places	55.56	17.99	
	Three places	56.31	23.01	
	More than three places	62.89	7.57	
Times per week to receive dialysis	1 time	67.61	0.00	0.813
	2 times	59.49	18.14	
	3 times	59.89	23.56	
Go to dialysis accompanied	Yes	56.88	17.99	0.002*
	No	66.15	16.47	
Presence of health insurance	Yes	62.13	18.41	0.001*
	No	53.79	15.47	
Type of insurance	Social health insurance	61.69	18.02	0.855
	Private insurance	66.93	24.11	
	Other	67.90	0.00	
Have you been hospitalized before or after the beginning of dialysis	Yes	61.35	18.61	0.971
	No	59.30	18.17	

^a^Participants were allowed to select more than one choice, that is why the analysis was performed as 0/1 for each choice

*significant p-value < 0.05

### Factors that correlate with the KDQOL components

A correlation matrix was conducted to examine the relationships between the cost of illness, the number of hospitalizations, and the duration of hospitalization against the scales of quality of life. The total cost of dialysis was significantly positively correlated with the burden of kidney disease (*r* = 0.203, *p* < 0.05). There was a significant, weak negative correlation between the number of hospitalizations and the following: SF-12 Mental Component (*r* = -0.249), burden of kidney disease (*r* = -0.330), effects of kidney disease (*r* = -0.303), and Kidney Disease Component Summary (*r* = -0.247; all *p* < 0.05) (Table S2).

### Kidney disease quality of life scale descriptive statistics

A 36-item KDQOL (KDQOL-36) was used to generate summary scores for the Physical Component Summary (PCS) and Mental Component Summary (MCS), with means ± SDs of 40.17 ± 9.01 and 47.10 ± 9.86, respectively. Additionally, the overall health scale score had a median of 60 and IQR of 30, while the patient satisfaction scale score was 66.67 (IQR = 17) (Table 5).

**Table 5:** Descriptive statistics of the kidney disease quality of life scale domain for the participants (*N* = 150)

Scale (number of items in scale)	Median	IQR
Symptom/problem list (12)	70.45	28
Effects of kidney disease (8)	71.65	31
Burden of kidney disease (4)	31.25	38
Work status (2)	0.00	50
Cognitive function (3)	86.67	33
Quality of social interaction (3)	93.33	20
Sexual function (2)	100.00	25
Sleep (4)	72.50	38
Social support (2)	100.00	33
Dialysis staff encouragement (2)	100.00	25
Overall health (1)	70.00	30
Patient satisfaction (1)	66.67	17
Physical functioning 10)	55.00	45
Role limitations–physical (4)	50.00	100
Pain (2)	66.25	65
General health (5)	60.00	30
Emotional well-being (5)	72.00	32
Role limitations–emotional (3)	100.00	100
Social function (2)	50.00	63
Energy/fatigue (4)	50.00	30
Kidney Disease Component Summary	59.52	59.52
	**Mean**	**SD**
SF-12 Physical Health Component	40.17	9.01
SF-12 Mental Health Component	47.10	9.86

IQR = Interquartile range

SD = Standard deviation

### Regression model for the SF-12 Physical Component

The general multiple regression model for the SF-12 Physical Component included factors with a p-value of < 0.25. It included age groups, gender, employment status, presence of hypertension, autoimmune disease, history of renal impairment, dialysis accompaniment, and history of hospitalization. The model was statistically significant (*p* = 0.004), with an R-squared value of 0.170. Among these variables, only age groups were significantly associated with the SF-12 Physical Component (*p* = 0.023) (Table S3).

### Regression model for Kidney Disease Component Summary

This analysis included factors with a p-value of < 0.25. The model included residency, educational level, stopping work due to renal failure, presence of hypertension, duration of renal failure, dialysis accompaniment, health insurance status, and frequency of hospitalization. The model was statistically significant (*p* = 0.048), with an R² value of 0.195. However, none of the included variables were significantly associated with the Kidney Disease Component Summary (Table S4).

## Discussion

Patients with end-stage kidney disease (ESKD) frequently experience significantly reduced quality of life (QOL) due to high mortality risk and adverse clinical outcomes [21–23]. This study aimed to identify modifiable factors influencing QOL to guide interventions for ESKD patients. Therefore, we used the Kidney Disease and Quality of Life-Short Form (KDQOL-SF™) to assess the quality of life for end-stage kidney disease patients in Khartoum State, Sudan, and linked with various sociodemographic and clinical characteristics in 150 participants.

While our findings on the impaired physical and mental health components align with global trends [24, 25], they provide the first evidence of this burden in Sudan’s unique healthcare context. More than half of our participants reported a duration of kidney failure of ≤ three years. A study conducted in Pakistan also reported a significant proportion of participants having a duration of kidney failure within a similar range of 2–4 years [24], indicating a comparable duration of kidney failure across these populations.

Hypertension and diabetes were the leading comorbidities, mirroring Kenyan data where hypertension was not only the most prevalent comorbidity but also the most prevalent underlying cause of kidney failure [25]. In contrast, a recently conducted study in Pakistan reported that diabetes was the comorbidity most prevalently associated with stage 5 CKD [24]. Comorbidities are known to impact ESKD patients’ survival and hospitalization rates [26]. Notably, a study concerning symptom management in CKD patients reported that patients on dialysis had a variety of symptoms that may be associated with their underlying ESKD, as well as various comorbidities, such as diabetes, hypertension, social situations, financial stresses, and/or multiple medications [27]. Over 85% of participants had health insurance, similar to rates reported in India [28]. Health insurance is intended to increase the accessibility and affordability of healthcare services. This approach becomes particularly crucial amidst the rising costs of health services. Therefore, a lack of insurance increases the economic burden on the patients and their families [29]. In this context, a study conducted in America revealed that people with CKD who do not have enough insurance pay a large amount out of pocket since they need continuous medical care throughout the year [30].

In terms of the burden of kidney disease, the present study revealed a relatively high burden compared to another study conducted in southern Kerala, India, which reported a lower burden on the kidney disease subscale [28]. These variations in the magnitude of the kidney disease burden may be attributed to the discrepancy in socioeconomic status between the different study populations, as there is an established association between low income and the progression of chronic kidney disease [31]. However, generally, these low scores may be attributed to factors such as adverse drug reactions, hospital stays, disrupted social life, and restricted diets that may increase the disease burden.

Cognitive function scores were higher in our study than in Indian study [28], suggesting preserved cognition despite ESKD, but a selection bias could not be excluded.

The Physical Component Summary (PCS) score in our study was low, aligning with findings from Kenya and India, which also demonstrated decreased physical functioning among patients with kidney disease [25, 28]. Low PCS scores likely reflect chronic symptoms, physical activity limitations, pain or discomfort, and diminished overall well-being. In the present study, PCS was significantly associated with employment status but not with education level. This finding contrasts with a study conducted in India, which found that higher education was associated with better PCS scores [28]. It also opposes the results of another Indian study, which identified significant associations between unemployment, illiteracy, and lower PCS scores [32]. Furthermore, the latter study highlighted unemployment and age as key predictors of PCS [32].

On the other hand, the Mental Component Summary (MCS) score was higher than the Physical Component Summary (PCS) score in both the present study and the Indian study [10]. This may be attributed to patients’ psychological adjustment to physical challenges. This moderate level of mental well-being among ESKD patients is consistent with findings from a study conducted in Kenya [25].

Diabetes was associated with higher Mental Component Summary (MCS) scores. However, we did not find any other significant factors associated with MCS scores.

Age has also been reported as a significant factor influencing MCS scores [28, 33]. For instance, research conducted in the United Kingdom found that older patients had higher MCS scores compared to younger patients, suggesting that older individuals may cope better emotionally with their kidney disease [33]. Conversely, another study from India [28] reported that younger patients had better MCS scores than older patients. Similarly, in Kenya [25], although the differences in MCS scores across age groups were not statistically significant, younger patients tended to have higher MCS scores than older ones. The older patients in the Kenyan study were more prone to emotional distress due to the heavy financial burden of dialysis, especially since many were unemployed. However, several other studies have shown that younger patients generally experience a better quality of life [34, 35].

We found no significant connection between having health insurance and the Physical or Mental Component Summary (PCS or MCS) scores. However, research from Nepal [36] did identify a strong link between having health insurance and both PCS and MCS scores. This suggests that health insurance may not cover the full range of needed services or address systemic healthcare challenges, limiting its effect on physical and mental health outcomes. Additionally, patients may face other barriers, such as inadequate healthcare infrastructure or financial constraints, which could undermine the potential benefits of insurance on their overall health. Moreover, factors such as cultural background, economic status, emotional well-being, access to medical care, and spiritual beliefs can significantly shape how individuals perceive their health and illness, potentially overshadowing the benefits of insurance.

We also observed that the Kidney Disease Component Summary (KDCS) score was notably higher than both the PCS and MCS scores. This suggests that patients generally experience fewer issues specifically related to kidney disease, perceive a lower burden from the disease, and thus report a better quality of life in this area.

Univariate analysis linked residence, working cessation due to the disease, high blood pressure, health insurance, and dialysis accompaniment to higher KDCS scores, but multivariate analysis showed no significant associations. In our study, factors such as place of residence, employment status, comorbidities, presence of companions for dialysis, and health insurance were significantly related to quality of life outcomes. This differs from findings in India [28] and Nepal [36], where older age, female gender, and lower education levels were associated with poorer quality of life scores. Additionally, we observed that the total cost of dialysis was positively correlated with the burden of kidney disease score, while the number of hospitalizations was negatively correlated with this score. This aligns with research from Japan, which found a strong association between kidney disease quality of life (KDQOL) and hospitalization in patients with end-stage kidney disease [10]. Moreover, several studies have shown that health-related quality of life (HRQOL) is a predictor of both mortality and hospitalization in dialysis patients [36, 37]. These findings suggest that the impact of factors such as healthcare costs and hospitalization on quality of life may differ across settings and highlight the importance of considering various contextual factors when evaluating quality of life outcomes.

This study is one of the first to assess health-related quality of life (HRQOL) in patients with end-stage kidney disease (ESKD) in Sudan using the validated KDQOL-SF™ 1.3 instrument. The inclusion of 13 renal centers across Khartoum State improves the representativeness of the findings. Data were collected through face-to-face interviews by trained medical students, enhancing the accuracy and completeness of responses.

However, several limitations should be considered. The cross-sectional design restricts the ability to infer causality between HRQOL and associated factors. The study did not explore the association between HRQOL and biochemical or laboratory parameters, which are important clinical indicators in ESKD. Cultural differences might influence how patients perceive and report quality of life, potentially affecting score interpretation.

## Conclusion

This study provides valuable insights into the quality of life (QOL) of patients with end-stage kidney disease (ESKD) in Khartoum State, Sudan. Both physical and mental health domains were significantly impaired in the studied ESKD population, aligning with global trends in dialysis care. The lowest scores were observed for disease burden and work status.

To improve the quality of life for ESKD patients in Sudan, it is recommended to enhance patient access to specialized healthcare services, strengthen health insurance coverage, and address the burden of comorbidities such as hypertension and diabetes. Promoting employment opportunities for patients, especially those with ESKD, can help alleviate the physical and mental impact of the disease. Additionally, increasing awareness about the importance of social support and dialysis accompaniment may improve patients’ overall well-being. It is also essential to reduce the financial burden associated with dialysis treatments through policy interventions and subsidies. Further research is needed to explore the unique socio-cultural factors affecting patients’ quality of life. Future studies should include matched controls and biochemical data to clarify causal relationships. More research on the disease burden is also essential to guide effective, tailored healthcare strategies.

## Electronic supplementary material

Below is the link to the electronic supplementary material.

Supplementary Material 1

## Data Availability

The datasets used and/or analysed during the current study are available from the corresponding author on reasonable request.

## Abbreviations

ESKD: End-stage kidney disease
HRQOL: Health-Related Quality of Life
KDQL-SF: Kidney Disease Quality of Life Short Form
CKD: Chronic Kidney Disease
eGFR: estimated Glomerular Filtration Rate
GFR: Glomerular Filtration Rate
RRT: Renal Replacement Therapy
SDG: Sudanese Pound
STROBE: Strengthening the Reporting of Observational Studies in Epidemiology
QOL: Quality of Life
PCS: Physical Component Summary
MCS: Mental Component Summary

## References

[1] Vaidya S, Aeddula N. Chronic Kidney Disease [Internet]. Nih.gov. StatPearls Publishing; 2022. Available from: https://www.ncbi.nlm.nih.gov/books/NBK535404/30571025

[2] Cleveland Clinic. Chronic Kidney Disease [Internet]. Cleveland Clinic. 2023. Available from: https://my.clevelandclinic.org/health/diseases/15096-chronic-kidney-disease

[3] Benjamin O, Lappin SL. end-stage kidney disease [Internet]. National Library of Medicine. StatPearls Publishing; 2023. Available from: https://www.ncbi.nlm.nih.gov/books/NBK499861/

[4] Temesgen Fiseha, Osborne NJ. Burden of end-stage kidney disease of undetermined etiology in Africa. Ren Replace Therapy. 2023;9(1).

[5] Mbeje PN, Mtshali N. Perceived predictors of quality of life in patients with end-stage kidney disease on dialysis. Curationis. 2021;44(1). 10.4102/curationis.v44i1.2251 PMC8517801 34636621

[6] Banaga ASI, Mohammed EB, Siddig RM, Salama DE, Elbashir SB, Khojali MO et al. Causes of end stage kidney failure among haemodialysis patients in Khartoum State/Sudan. BMC Research Notes [Internet]. 2015;8(1). Available from: https://bmcresnotes.biomedcentral.com/articles/10.1186/s13104-015-1509-x 10.1186/s13104-015-1509-x PMC4589074 26419536

[7] Elamin S, Obeid W, Abu-Aisha H. Renal replacement therapy in sudan, 2009. Arab J Nephrol Transplantation. 2010;3(2).

[8] Elkhidir HAHOA, Elawad SO et al. Ola Hatim Elniema, Mustafa Khalid Khalid, Lina Sameer Altayib,. The burden of end-stage renal disease in Khartoum, Sudan: cost of illness study. Journal of Medical Economics. 2024;27(1):1–36. 10.1080/13696998.2024.2320506 38390791

[9] Sitlinger A, Yousuf Zafar S. Health-Related Quality of Life. Surgical oncology clinics of North America [Internet]. 2018;27(4):675–84. Available from: https://www.ncbi.nlm.nih.gov/pmc/articles/PMC6428416/10.1016/j.soc.2018.05.008 PMC6428416 30213412

[10] Fukuhara S, Lopes AA, Bragg-Gresham JL, Kurokawa K, Mapes DL, Akizawa T, et al. Health-related quality of life among dialysis patients on three continents: the Dialysis outcomes and practice patterns study. Kidney Int. 2003;64(5):1903–10. 14531826 10.1046/j.1523-1755.2003.00289.x

[11] Al-Rajhi W, Al Salmi I. Quality of life and Health-related quality of life in patients with End-stage kidney disease undergoing hemodialysis: A literature review. Saudi J Kidney Dis Transplantation. 2022;33(Suppl 2):S184–230. 10.4103/1319-2442.384191 37675749

[12] Tang E, Bansal A, Novak M, Mucsi I. Patient-Reported outcomes in patients with chronic kidney disease and kidney Transplant—Part 1. Front Med. 2018;4:254. 10.3389/fmed.2017.00254 PMC5775264 29379784

[13] Yang F, Griva K, Lau T, Vathsala A, Lee E, Ng HJ, et al. Health-related quality of life of Asian patients with end-stage kidney disease (ESKD) in Singapore. Qual Life Res. 2015;24(9):2163–71. 25800727 10.1007/s11136-015-0964-0

[14] Kaballo B, Idris M, Alhaj H, Gadour M. Psychological disorders and quality of life among Sudanese Dialysis patients and renal transplant recipients. Sudan J Med Sci. 2010;5(1).

[15] von Elm E, Altman DG, Egger M, Pocock SJ, Gøtzsche PC, Vandenbroucke JP. The strengthening the reporting of observational studies in epidemiology (STROBE) statement: guidelines for reporting observational studies. Lancet. 2007;370(9596):1453–7. 18064739 10.1016/S0140-6736(07)61602-X

[16] Ahmet, Yılmaz. Cochran 1977 Sampling Techniques Third Edition [Internet]. Academia.edu. 2015. Available from: https://www.academia.edu/29684662/Cochran_1977_Sampling_Techniques_Third_Edition

[17] Bello AK, Levin A, Lunney M, Osman MA, Ye F, Ashuntantang G, et al. Global kidney health atlas: A report by the international society of nephrology on the global burden of End-stage kidney disease and capacity for kidney replacement therapy and Conservative care across world countries and regions. Brussels: International Society of Nephrology; 2019.

[18] Hays R, Kallich J, Mapes D, Coons S, Amin N, Carter W, Kamberg C. Kidney Disease Quality of Life-Short Form (KDQOL-SF™), version 1.3: a manual for use and scoring. 1997, Santa Monica, CA, USA, RAND (P-7994).

[19] Hays RD, Kallich JD, Mapes DL, Coons SJ, Carter WB. Development of Kidney Disease Quality of Life (KDQOL) Instrument. Quality of Life Research. 1994; 3: 329–338. 10.1007/BF00451725 7841967

[20] Mapes DL, Bragg-Gresham JL, Bommer J, Fukuhara S, McKevitt P, Wikström B, Lopes AA. Health-related quality of life in the Dialysis outcomes and practice patterns study (DOPPS). Am J Kidney Dis. 2004;44(5 Suppl 2):54–60. 15486875 10.1053/j.ajkd.2004.08.012

[21] Lowrie EG, Curtin RB, LePain N, Schatell D. Medical outcomes study short form-36: a consistent and powerful predictor of morbidity and mortality in dialysis patients. Am J Kidney Dis. 2003;41(6):1286–92. 12776282 10.1016/s0272-6386(03)00361-5

[22] Kalantar-Zadeh K, Kopple JD, Block G, Humphreys MH. Association among SF36 quality of life measures and nutrition, hospitalization, and mortality in Hemodialysis. J Am Soc Nephrol. 2001;12(12):2797–806. 11729250 10.1681/ASN.V12122797

[23] Mittal SK, Ahern L, Flaster E, Maesaka JK, Fishbane S. Self-assessed physical and mental function of haemodialysis patients. Nephrol Dialysis Transplantation. 2001;16(7):1387–94. 10.1093/ndt/16.7.1387 11427630

[24] Butt MD, Ong SC, Butt FZ, Sajjad A, Rasool MF, Imran I et al. Assessment of Health-Related Quality of Life, Medication Adherence, and Prevalence of Depression in Kidney Failure Patients. International Journal of Environmental Research and Public Health [Internet]. 2022;19(22):15266. Available from: https://www.mdpi.com/1660-4601/19/22/15266 10.3390/ijerph192215266 PMC9690334 36429988

[25] Kamau. The determinants of health related quality of life of patients on maintenance haemodialysis at Kenyatta National Hospital, Kenya. East African medical journal [Internet]. 2014 [cited 2024 Aug 17];91(10). Available from: https://pubmed.ncbi.nlm.nih.gov/26862616/26862616

[26] Cha J, Han D, Health-Related Quality of Life Based on Comorbidities Among Patients with End-Stage Renal Disease. Osong Public Health and Research Perspectives [Internet]. 2020;11(4):194–200. Available from: https://www.ncbi.nlm.nih.gov/pmc/articles/PMC7442444/10.24171/j.phrp.2020.11.4.08 PMC7442444 32864310

[27] Cabrera VJ, Hansson J, Kliger AS, Finkelstein FO. Symptom Management of the Patient with CKD: The Role of Dialysis. Clinical Journal of the American Society of Nephrology [Internet]. 2017;12(4):687–93. Available from: https://cjasn.asnjournals.org/content/12/4/687.short 10.2215/CJN.01650216 PMC5383375 28148557

[28] Manju L, Joseph J. Health-related quality of life among patients undergoing hemodialysis in a tertiary center in South Kerala. Clinical Epidemiology and Global Health [Internet]. 2024;26(101547):101547. Available from: https://www.sciencedirect.com/science/article/pii/S2213398424000435

[29] Tolbert J, Cervantes S, Bell C, Damico A. Key facts about the uninsured population [Internet]. Kaiser Family Foundation. 2024. Available from: https://www.kff.org/uninsured/issue-brief/key-facts-about-the-uninsured-population/

[30] Acquah I, Valero-Elizondo J, Javed Z, Ibrahim HN, Patel KV, Ryoo Ali HJ, et al. Financial hardship among nonelderly adults with CKD in the united States. Am J Kidney Dis. 2021;78(5):658–68. 34144103 10.1053/j.ajkd.2021.04.011

[31] Zeng X, Liu J, Tao S, Hong HG, Li Y, Fu P. Associations between socioeconomic status and chronic kidney disease: a meta-analysis. J Epidemiol Commun Health. 2018;72(4):jech–2017. 10.1136/jech-2017-209815 29437863

[32] Priyamvada P, Manavalan M, Majumdar A, Harichandra Kumar K. Assessment of health-related quality of life and its determinants in patients with chronic kidney disease. Indian J Nephrol. 2017;27(1):37. 28182041 10.4103/0971-4065.179205 PMC5255988

[33] Surtees PG, Wainwright NWJ, Khaw KT, Day NE. Functional health status, chronic medical conditions and disorders of mood. The British Journal of Psychiatry [Internet]. 2003;183(4):299–303. Available from: https://www.cambridge.org/core/journals/the-british-journal-of-psychiatry/article/functional-health-status-chronic-medical-conditions-and-disorders-of-mood/CDCC897D0A108B269A97F0B8F7C13B80 10.1192/bjp.183.4.299 14519607

[34] Zyoud SH, Daraghmeh DN, Mezyed DO, Khdeir RL, Sawafta MN, Ayaseh NA et al. Factors affecting quality of life in patients on haemodialysis: a cross-sectional study from Palestine. BMC Nephrol. 2016;17(1). 10.1186/s12882-016-0257-z PMC4847206 27117687

[35] Mousa I, Ataba R, Al-ali K, Alkaiyat A, Zyoud SH. Dialysis-related factors affecting self-efficacy and quality of life in patients on haemodialysis: a cross-sectional study from Palestine. Ren Replace Therapy. 2018;4(1).

[36] Mahato SKS, Apidechkul T, Sriwongpan P, Hada R, Sharma GN, Nayak SK et al. Factors associated with quality of life among chronic kidney disease patients in nepal: a cross-sectional study. Health Qual Life Outcomes. 2020 June 29;18(1). 10.1186/s12955-020-01458-1 PMC7325283 32600360

[37] Pei M, Aguiar R, Pagels AA, Heimbürger O, Stenvinkel P, Bárány P et al. Health-related quality of life as predictor of mortality in end-stage renal disease patients: an observational study. BMC Nephrol. 2019;20(1). 10.1186/s12882-019-1318-x PMC6489294 31035977

