# Supplementary material for: Exploring the quality of life of end-stage kidney disease patients in Khartoum State, Sudan: a multicenter cross-sectional study
Source: BMC Nephrol. 2025 Jul 1;26:336. doi: 10.1186/s12882-025-04257-2 (PMC12218816; doi:10.1186/s12882-025-04257-2)
Supplement: Supplementary file 1 — Supplementary Material 1 [file 12882_2025_4257_MOESM1_ESM.docx]

**Table S1**: Sample distribution of participants in different renal centers

| Renal centers | Registered per  center | Weight | Sample from each  center | Interval |
| --- | --- | --- | --- | --- |
| **Alwaledeen** | 183 | 0.090 | 14 | 13 |
| **Tropical diseases** | 132 | 0.065 | 10 | 19 |
| **Alnaw** | 134 | 0.066 | 10 | 18 |
| **Ombada** | 157 | 0.078 | 12 | 16 |
| **Chinese friendship** | 153 | 0.076 | 11 | 16 |
| **Military hospital** | 119 | 0.059 | 9 | 21 |
| **Academy** | 180 | 0.089 | 13 | 14 |
| **Elshahida Salma** | 195 | 0.097 | 14 | 13 |
| **Bashaier** | 104 | 0.051 | 8 | 24 |
| **Ibnsina** | 225 | 0.111 | 17 | 11 |
| **Elsafia** | 207 | 0.102 | 15 | 12 |
| **Renal transplant association** | 126 | 0.062 | 9 | 20 |
| **Ahmed Gasim** | 105 | 0.052 | 8 | 23 |
| **The total** | **2020** |  | **150** |  |

**Table S2:** Correlation matrix for the cost of illness, frequency of hospitalizations, and duration of hospitalization against the quality of life scales (**N=150**)

| **Variables** | **SF-12**  **Physical Component** | **SF-12**  **Mental Component** | **Burden of**  **kidney disease** | **Symptom/ problem**  **list** | **Effects of kidney disease** | **Kidney Disease Component Summary** |
| --- | --- | --- | --- | --- | --- | --- |
| Total cost for the dialysis | 0.117 | 0.013 | 0.203* | -0.107 | 0.121 | 0.096 |
| Frequency of hospitilization | -0.097 | -0.249* | -0.330* | 0.042 | -0.303* | -0.247* |
| Duration of hospitalization | -0.192 | -0.093 | -0.062 | 0.047 | -0.198 | -0.166 |

*significant p-value<0.05

**Table S3:** Multiple regression for sociodemographic, clinical, and dialysis center characteristics regarding the SF-12 Physical Component **(N=150**)

| Variables | Unstandardized Coefficients | | Standardized Coefficients | T | p-value | 95% Confidence Interval for B | |
| --- | --- | --- | --- | --- | --- | --- | --- |
|  | B | Std. Error | Beta |  |  | Lower  Bound | Upper Bound |
|  |  |  |  |  |  |  |  |
| (Constant) | 20.253 | 14.777 |  | 1.371 | .173 | -9.011 | 49.518 |
| Age groups | -1.313 | .571 | -.208 | -2.297 | .023* | -2.444 | -.181 |
| Gender | -.871 | 1.701 | -.047 | -.512 | .610 | -4.239 | 2.497 |
| Employment Status | -.466 | .259 | -.168 | -1.800 | .074 | -.980 | .047 |
| Presence of hypertension as comorbidity | 1.474 | 1.747 | .075 | .844 | .401 | -1.985 | 4.933 |
| Presence of Autoimmune disease as comorbidity | 9.272 | 6.702 | .118 | 1.384 | .169 | -4.001 | 22.545 |
| Presence of a history of renal impairment | 2.031 | 1.535 | .113 | 1.323 | .188 | -1.008 | 5.071 |
| Go to dialysis accompanied | 2.721 | 1.639 | .149 | 1.660 | .099 | -.524 | 5.967 |
| Have you been hospitalized before? | .304 | 1.699 | .017 | .179 | .858 | -3.061 | 3.669 |
| **Dependent Variable: SF-12 Physical** **Component** | | | | | | | |

*significant p-value<0.05

**Table S4:** Multiple regression for sociodemographic, clinical, and dialysis center characteristics regarding the Kidney Disease Component Summary **(N=150**)

| **Variables** | **Unstandardized Coefficients** | | **Standardized Coefficients** | **t** | **p-value** | **95.0% Confidence Interval for B** | | **Collinearity Statistics** | |
| --- | --- | --- | --- | --- | --- | --- | --- | --- | --- |
|  | **B** | **Std. Error** | **Beta** |  |  | **Lower Bound** | **Upper Bound** | **Tolerance** | **VIF** |
| (Constant) | 27.067 | 16.336 |  | 1.657 | 0.102 | -5.521 | 59.656 |  |  |
| Residency | 6.310 | 4.317 | 0.162 | 1.462 | 0.148 | -2.302 | 14.923 | 0.951 | 1.052 |
| Educational level | 2.018 | 2.227 | 0.104 | .906 | 0.368 | -2.425 | 6.460 | 0.877 | 1.140 |
| Have you stopped working due to renal failure? | 4.793 | 4.509 | 0.124 | 1.063 | 0.291 | -4.202 | 13.788 | 0.857 | 1.167 |
| Presence of hypertension | 6.839 | 4.552 | 0.170 | 1.503 | 0.138 | -2.241 | 15.919 | 0.914 | 1.094 |
| Duration of renal failure in years | 2.177 | 2.336 | 0.113 | .932 | 0.354 | -2.482 | 6.836 | 0.798 | 1.252 |
| Go to dialysis accompanied | 6.513 | 4.403 | 0.169 | 1.479 | 0.144 | -2.271 | 15.297 | 0.888 | 1.126 |
| Do you have health insurance? | -8.206 | 6.164 | -0.152 | -1.331 | 0.188 | -20.503 | 4.092 | 0.896 | 1.116 |
| Frequency of hospitilzation | -.264 | .602 | -0.052 | -0.439 | 0.662 | -1.464 | 0.936 | 0.831 | 1.204 |
| **Dependent Variable:** **Kidney Disease Component Summary** | | | | | | | | | |
